# Exploring the Needs of Adolescents With Sickle Cell Disease to Inform a Digital Self-Management and Transitional Care Program: Qualitative Study

**DOI:** 10.2196/11058

**Published:** 2018-09-25

**Authors:** Yalinie Kulandaivelu, Chitra Lalloo, Richard Ward, William T Zempsky, Melanie Kirby-Allen, Vicky R Breakey, Isaac Odame, Fiona Campbell, Khush Amaria, Ewurabena A Simpson, Cynthia Nguyen, Tessy George, Jennifer N Stinson

**Affiliations:** 1 Department of Child Health Evaluative Sciences The Hospital for Sick Children Toronto, ON Canada; 2 Institute of Health Policy, Management & Evaluation University of Toronto Toronto, ON Canada; 3 Division of Haematology University Health Network Toronto, ON Canada; 4 Division of Pain and Palliative Medicine Connecticut Children's Medical Center University of Connecticut School of Medicine Hartford, CT United States; 5 Departments of Haematology/Oncology The Hospital for Sick Children Toronto, ON Canada; 6 Department of Pediatrics McMaster Children's Hospital Hamilton, ON Canada; 7 Department of Pediatrics McMaster University Hamilton, ON Canada; 8 Department of Paediatrics University of Toronto Toronto, ON Canada; 9 Department of Anesthesia and Pain Medicine The Hospital for Sick Children Toronto, ON Canada; 10 Department of Anesthesia Faculty of Medicine University of Toronto Toronto, ON Canada; 11 Department of Psychology The Hospital for Sick Children Toronto, ON Canada; 12 Division of Hematology/Oncology Children's Hospital of Eastern Ontario Ottawa, ON Canada; 13 Faculty of Medicine University of Ottawa Ottawa, ON Canada

**Keywords:** sickle cell, adolescent, cell phone, self-management, internet, qualitative research, needs assessment, transitional care

## Abstract

**Background:**

Accessible self-management interventions are critical for adolescents with sickle cell disease to better cope with their disease, improve health outcomes and health-related quality of life, and promote successful transition to adult health care services. However, very few comprehensive self-management and transitional care programs have been developed and tested in this population. Internet and mobile phone technologies can improve accessibility and acceptability of interventions to promote disease self-management in adolescents with sickle cell disease.

**Objective:**

The aim of this study was to qualitatively explore the following from the perspectives of adolescents, parents, and their health care providers: (1) the impact of sickle cell disease on adolescents to identify challenges to their self-management and transitional care and (2) determine the essential components of a digital self-management and transitional care program as the first phase to inform its development.

**Methods:**

A qualitative descriptive design utilizing audio-recorded, semistructured interviews was used. Adolescents (n=19, aged 12-19 years) and parents (n=2) participated in individual interviews, and health care providers (n=17) participated in focus group discussions and were recruited from an urban tertiary care pediatric hospital. Audio-recorded data were transcribed verbatim and organized into categories inductively, reflecting emerging themes using simple content analysis.

**Results:**

Data were categorized into 4 major themes: (1) impact of sickle cell disease, (2) experiences and challenges of self-management, (3) recommendations for self-management and transitional care, and (4) perceptions about a digital self-management program. Themes included subcategories and the perspectives of adolescents, parents, and health care providers. Adolescents discussed more issues related to self-management, whereas health care providers and parents discussed issues related to transition to adult health services.

**Conclusions:**

Adolescents, parents, and health care providers described the continued challenges youth with sickle cell disease face in terms of psychosocial impacts and stigmatization. Participants perceived a benefit to alleviating some of these challenges through a digital self-management tool. They recommended that an effective digital self-management program should provide appropriate sickle cell disease–related education; guidance on developing self-advocacy and communication skills; empower adolescents with information for planning for their future; provide options for social support; and be designed to be engaging for both adolescents and parents to use. A digital platform to deliver these elements is an accessible and acceptable way to address the self-management and transitional care needs of adolescents.

## Introduction

Sickle cell disease (SCD) is an inherited red blood cell disorder that predominantly affects individuals of African descent. Increased prevalence is also seen in Mediterranean, Caribbean, South and Central American, Arab, and East Indian populations [1]. SCD is caused by a genetic mutation that results in abnormal hemoglobin, which makes blood cells rigid and sickled in shape. These abnormal red cells can disrupt the flow of blood in small vessels, resulting in hypoxia-reperfusion injury throughout the body and its organs, causing episodes of painful vaso-occlusive crises and organ damage. Cerebral infarcts resulting in neurological and neurocognitive deficits are the most feared complications [2]. Negative consequences from recurrent pain in SCD include academic underachievement related to school absenteeism, limited physical activity, symptoms of depression and anxiety, and disruption of interpersonal relations [3,4].

Despite the suffering and burden associated with SCD, minimal research has focused on promoting disease self-management among adolescents with SCD, such as teaching skills to help teens to cope with symptoms and manage their chronic illness. Many youth with SCD leave pediatric care without adequate transition preparation and subsequently do not successfully transfer to adult health care services [5,6], which may put them at risk for higher morbidity and earlier mortality during early adulthood years [5-7]. Therefore, improving health-related quality of life and ensuring successful transition to adult health care through better disease self-management is critical [5,6,8,9].

Self-management interventions such as information-based material and cognitive behavioral therapies (CBTs) for SCD have shown promise in symptom reduction and improved health-related quality of life [8,10,11]. However, the vast majority of adolescents with chronic health conditions do not receive comprehensive self-management (eg, CBT) education due to lack of access to these therapies. This limited accessibility is a function of the direct and indirect costs associated with therapy as well as limited availability of trained professionals, particularly in nonurban areas [8]. There is a clear need to develop accessible and acceptable ways to deliver self-management and transitional care therapies to adolescents with SCD and their families.

Information and communication technologies offer an accessible platform for the delivery of health interventions for patients and families [12-16]. Access to Web-based health interventions eliminates geographic constraints, allows for anonymity, and provides 24-hour access to disease-related information and self-management strategies that may help patients feel more in control of managing their health problems and minimize feelings of isolation [12,13]. Digital interventions are treatments based on effective face-to-face interventions (eg, CBT) that are transformed for delivery via the internet with the goal of improved health outcomes. Previous efforts in developing interventions for adolescents with SCD have focused on medication adherence, symptom tracking, and CBT for pain management [17-20]. However, many of these programs are characterized by limited involvement of adolescents and their families in their development.

To date, no digital, comprehensive self-management and transitional care program has been developed and evaluated in terms of improved health outcomes (symptom reduction and improved transition readiness and health-related quality of life) for adolescents with SCD. Thus, the aims of this study were to qualitatively examine the following from the perspectives of adolescents, parents, and their health care providers (HCPs): (1) examine the impact of SCD on adolescents to identify challenges to their self-management and transitional care and (2) determine the key components of a digital program for SCD self-management and transitional care for adolescents.

## Methods

### Patient and Parent Selection

Adolescents, parents, and HCPs were purposively recruited from 1 large metropolitan tertiary care pediatric center in Toronto, Canada. Adolescents were eligible to participate if they were (1) 12 to 19 years old, (2) diagnosed with SCD, and (3) able to speak and read English and at least one of their parents was able to speak and read English. Adolescents were excluded if they had (1) severe cognitive impairments or (2) major medical or psychiatric concurrent illnesses, which precluded assessment of their self-management and transitional care needs. Parents were eligible to participate if they were able to speak and read English.

### Health Care Provider Selection

HCPs were eligible to participate if they (1) had worked in pediatric hematology, general pediatrics, and emergency medicine for at least 1 year at the time of the study and provided care to adolescents with SCD and (2) could speak and read English.

### Study Design

A descriptive qualitative design with semistructured, audiotaped individual interviews with adolescents with SCD and parents, and focus group interviews with HCPs, were undertaken to examine the impact of SCD on adolescents; to determine self-management and transitional care needs of adolescents with SCD; and to inform the development of a Web-enabled self-management and transitional care intervention [21,24]. Individual interviews were utilized to gain an appreciation of the perspectives of the individual adolescents and their parents, uninfluenced by the views of others. Focus group interviews were conducted with HCPs to capitalize on communication and shared interaction to generate data and gain insight into specific issues in more depth [22].

### Procedures

The local institutional research ethics board approved the study. The research assistant obtained consent, arranged an individual interview with each of the parent and adolescent participants, and asked them to complete questionnaires on demographics and on internet use. The research assistant completed a health information questionnaire to gather additional demographic and medical information from the adolescent’s chart. The research assistant conducted the individual interviews with adolescents and parents. Interview questions were based on a literature review, followed by pilot testing with 2 to 3 adolescents with SCD and their parents, and modified based on their feedback (see Textbox 1 for semistructured interview guide). Interviews began with a broad introductory question, followed by questions and probes to allow participants to elaborate on their experiences. Refreshments and a small honorarium were provided to compensate for participant time.

HCP participants were recruited using posters describing the study and information sessions at the pediatric center. After obtaining consent from HCPs, a mutually convenient time was set up for the focus group interviews once 5 to 7 participants were enrolled. HCPs were also asked to complete a questionnaire on demographics. Trained members of the research team moderated the focus group discussions among the HCPs. During and immediately after the focus group discussions took place, the interviewer detailed field notes on their impressions of participant responses and the interviewing process. All individual and focus group interviews were audio-recorded and transcribed verbatim.

### Data Analysis

All data from individual and focus group interviews and field notes were analyzed inductively using simple content analysis [23]. Transcribed data were managed using NVivo computer software program (QSR International). Demographic data were analyzed using descriptive statistics in Microsoft Excel and used to summarize the characteristics of the study participants. All data were read several times by 3 investigators (JNS, CN, and YK) to obtain an overall understanding, identify data codes, and ensure that all comments were carefully considered and included.

**Broad questions in semistructured interview guide.**

1. Can you tell me what it has been like for you/your son or daughter/adolescents to live with sickle cell disease (SCD)?
2. What do you think is important to know and do so that you/your son or daughter/adolescents can learn or manage (or take care of) your/their SCD?
3. What do you/your son or daughter/adolescents need to know about your/their SCD?
4. What do you/your son or daughter/adolescents need to know about in terms of how to manage or treat your/their SCD?
5. What do you/your son or daughter/adolescents need to know about drugs and other treatments and how they affect you/them?
6. Can you tell me what it is like for you/your son or daughter/adolescents when you/they come to the emergency department for a sickle cell crisis?
7. Can you tell me about how SCD affects the other aspects of your/your son or daughter/adolescents’ life?
8. What is it like (for your son or daughter/adolescents) to talk about your/their SCD to your/their doctors and nurses? Family? Friends? Teachers?
9. When you/your son or daughter/adolescents turn 18, you/they will be transferred from a pediatric hospital to an adult hospital. Have you thought about this? What do you think it will be like (for them)?
10. How do you think SCD will affect you/your son or daughter/adolescents’ in the future?
11. How have you/your son or daughter/adolescents learned about your/their SCD and how to manage and treat it?
12. If you/your son or daughter/adolescents had to tell someone else about what it’s like to have SCD and how to deal with it what would you/they tell them?
13. What do you think it would be like (for your son or daughter/adolescents) to learn more about your/their SCD from a web site made just for teens and young adults with SCD and their families?
14. Is there anything else you would like to tell us about what you think is important to know and do so that you/your son or daughter/adolescents can learn to take care of your/their SCD better?

As data were entered into the analyses, codes continued to be generated, until there were no new data that could not be categorized under existing codes. Codes were combined into higher-level themes, and the themes were checked against coded extracts and the entire dataset [23,24]. Disagreements were addressed through discussion and consensus of all investigators.

## Results

### Participant Characteristics

A total of 19 adolescents, 2 parents, and 17 HCPs were recruited from March 2012 to August 2012. Adolescents and parents individually participated in the semistructured interviews, and HCPs participated in focus groups. Participant demographics, medical information, and internet and computer use are summarized in Tables 1 and 2. HCP demographics information is summarized in Table 3. Of the 18 adolescents who responded to the internet and computer use questionnaire, 94% (17/18) had a computer with internet access and were either “comfortable” or “very comfortable” using the internet.

### Self-Management and Transitional Care Needs

The perspectives of participants were categorized into 4 major themes: “impact of sickle cell disease,” “experiences and challenges of self-management of SCD,” “recommendations for self-management and transitional care,” and “perceptions of digital self-management program.” Subthemes for each of the major themes are summarized below and in Textbox 2; additional quotations illustrating the themes and subthemes can be found in Multimedia Appendix 1.

#### Impact of Sickle Cell Disease

##### Uncertainty of Sickle Cell Disease

Adolescents, parents, and HCPs all described the challenges of managing the uncertainty of SCD. Parents and HCPs described the uncertainty of complications such as strokes and downstream consequences associated with them such as cognitive and vision impairments. Adolescents worried about the uncertainty of vaso-occlusive crises and whether any feeling of pain would escalate into a crisis and whether it would affect school or activities with friends. HCPs described the emotional impact this had on adolescents, resulting in frustration and a feeling of loss of control over their lives.

##### Impact on Developing Peer Relationships

Adolescents reported frequently missing out on activities with friends, such as swimming and playing outside, along with school events due to appointments and crises and described feeling different from their peers. Adolescents reported that this affected their ability to create and maintain friendships, especially when they were younger. Adolescents and parents described experiences of being bullied for seeming different from their peers; however, some adolescents and parents found that as they grew older, it became easier to make friends who were considerate of their condition. Other adolescents preferred not to speak with friends or teachers about their disease and kept it to themselves. Parents and HCPs found that children would also forgo strategies to prevent a crisis around their friends because they wanted to fit in. HCPs discussed the challenges of managing SCD during adolescence when youth were going through many different transitions in their lives and were trying hard to fit in among their peers.

##### Academic Impact

Adolescents discussed missing many days of school, especially with crises and appointments, resulting in missed homework, lessons, tests, and projects and catching up with missed schoolwork. Keeping up with missed schoolwork was also a source of stress described by adolescents, parents, and HCPs. Adolescents described how keeping up with missed schoolwork was a source of stress that caused vaso-occlusive crises. All 3 groups noted that teachers needed education on the disease so that they would not be skeptical of adolescents’ symptoms or absences.

##### Sickle Cell Disease Is Stigmatizing

Adolescents described difficulties in explaining their disease to others and dealing with negative responses from others, which was echoed by HCPs. Negative responses from peers were often exacerbated by negative responses and misunderstanding of their condition among teachers. HCPs described the racial stigma of SCD among HCPs in emergency departments. Other HCPs described the disease-related stigma adolescents faced when they sought care, often being labeled as *drug-seekers*. HCPs also described the cultural stigma perceived to exist among some of the ethnic groups who typically inherited SCD.

#### Experience and Challenges of Self-Management

##### The Internet Is a Source of Sickle Cell Disease–Related Education

Adolescents learned about SCD from HCPs, parents, and frequently from the internet. However, using the internet to learn more did not always meet their needs in a developmentally appropriate way. Parents used the internet as a resource and informed their children about the disease. They knew which websites were more reputable and accurate than others to get their information.

##### Managing Emergency Department Visits

Adolescents and parents highlighted a need for further awareness and education among emergency department staff on SCD and its management. HCPs described the challenges adolescents would face in attending emergency departments that were not familiar with them or SCD; this often resulted in delays in pain management. Adolescents and parents found that they waited too long for pain management at certain emergency departments and thus preferred visiting hospitals where emergency departments had implemented an SCD protocol or who had experience managing SCD vaso-occlusive crises.

##### Self-Management Is a Joint Effort

Adolescents believed managing their disease was primarily their responsibility along with their parents, HCPs, and sometimes friends, and adolescents described it as a joint effort between everyone. They knew as they grew older, self-management was ultimately their own responsibility, but their parents were still involved in reminding them to take their medication, make appointments, bring them to appointments, knowing when to go to the hospital, and to advocate for them when needed. Adolescents said that they were starting to take their own medication and taking preventative measures against crises. Having supportive friends and family, communicating with friends with SCD, and taking charge of their disease were all strategies in their self-management. Parents said that they were still mostly managing their teen’s disease but that they were trying to step back and give adolescents opportunities to take charge of their care.

##### Lack of System Level Supports

Parents described the financial challenges of managing SCD. They took time off from work for hospital visits and emergency department admissions and felt worried when they had to go to work while their child was still in the hospital. HCPs discussed the lack of dedicated financial and practical supports for SCD in the province. Another challenge to accessing existing supports was the lack of awareness among families, often due to financial and communication barriers due to a largely immigrant population.

**Table 1:** Demographic characteristics of adolescents.

Characteristic		Adolescents (N=19)^a^
Age in years, mean (SD)		15 (1.9)
**Sex, n (%)**		
	Female	12 (66)
	Male	6 (33)
**School grade, n (%)**		
	Grade 7	1 (12)
	Grade 8	6 (33)
	Grade 9	2 (11)
	Grade 10	1 (12)
	Grade 11	5 (27)
	Grade 12	2 (11)
	Other	1 (12)
**Diagnosis^b^, n (%)**		
	Sickle cell disease hemoglobin SS	14 (77)
	Sickle cell disease hemoglobin SC	3 (16)
**In the past 6 months, how many times have you..., mean (SD)**		
	Been admitted to the hospital^c^	2 (2.6)
	Been to an emergency department	1.05 (1.8)
**Current prescribed medications for sickle cell disease management^d^, n (%)**		
	Acetaminophen	1 (5)
	Morphine	3 (16)
	Folic acid	5 (27)
	Hydroxyurea	5 (27)
	Penicillin	1 (5)
	Deferasirox	8 (44)
	Calcium	1 (5)
	Aspirin	4 (22)
	Salbutamol	1 (5)
	Fluticasone	1 (5)

^a^One participant did not respond to the entire questionnaire.

^b^N=2 did not respond; participants could list more than 1 diagnosis.

^c^N=1 did not respond.

^d^N=1 did not respond; participants could list more than 1 medication.

**Table 2:** Computer use of adolescents.

Characteristic		Adolescents (N=18)^a^
**Do you use a computer at home?, n (%)**		
	Yes	17 (94)
	No	1 (5)
**In 1 week, how many hours do you use the computer?, n (%)**		
	Not at all	0 (0)
	<1 hour	1 (5)
	1-2 hours	2 (11)
	2-3 hours	0 (0)
	3-4 hours	4 (22)
	4-5 hours	1 (5)
	5-6 hours	3 (16)
	6-7 hours	3 (16)
	>7 hours	4 (22)
**Please circle the number that goes with how comfortable you feel using a computer, n (%)**		
	Not at all comfortable	0 (0)
	A little comfortable	0 (0)
	Comfortable	9 (50)
	Very comfortable	9 (50)

^a^One participant did not respond to the questionnaire.

**Table 3:** Demographic characteristics of health care providers.

Characteristic		Health care providers (N=17)
Age in years, mean (SD)		38.6 (7.2)
**Sex, n (%)**		
	Female	14 (82)
	Male	3 (17)
**Profession, n (%)**		
	Staff hematologist/oncologist	1 (5)
	Fellow	2 (11)
	Resident	3 (17)
	Staff nurse	4 (23)
	Advanced practice nurse	3 (17)
	Psychologist	2 (11)
	Other	4 (23)
Number of years of health professional experience (including training), mean (SD)		17.6 (8.4)
Number of years of pediatric hematology/oncology experience, mean (SD)		8 (7.9)
**Are you a parent?, n (%)**		
	Yes	6 (35)
	No	11 (64)

**Summary of themes and subthemes.**

Self-management and transitional care needs of adolescents with sickle cell disease (SCD)

1. Impact of SCD
  - Uncertainty of SCD
  - Impact on developing peer relationships
  - Academic impact
  - SCD is stigmatizing
2. Experiences and challenges of self-management
  - The internet is a source of SCD-related education
  - Managing emergency department visits
  - Self-management is a joint effort
  - Lack of system-level supports
3. Recommendations for self-management and transitional care
  - Information to cope with and live with SCD
  - Self-advocacy and communication
  - Social support
  - Information for future planning
  - Transition is not one-size fits all
4. Perceptions about digital self-management program
  - Facilitating transition care
  - Accessibility
  - Effectively engaging adolescents and families

#### Recommendations for Self-Management and Transitional Care

##### Information to Cope With and Live With Sickle Cell Disease

All groups voiced that understanding information about SCD was at the core of self-management. Adolescents wanted to know how they got the disease, symptoms, diagnosis, treatment options, different types of the disease, genetic components, and strategies for preventing vaso-occlusive crises. Adolescents were interested in knowing more about their limits to physical activity and how they could make the most of being involved in activities without triggering a crisis. They discussed the importance of understanding the steps to take during a crisis and how to manage it in terms of medication and care. Parents and HCPs believed adolescents needed to understand the consequences of nonadherence to medication. HCPs suggested that empowering adolescents with strategies for preventing crises could help them cope with the uncertainty of SCD.

##### Self-Advocacy and Communication

Adolescents found that explaining SCD to teachers and peers was difficult because they did not know the best way to describe the disease or answer their questions. In addition, HCPs and adolescents cited a need for learning strategies to communicate about their disease to peers, teachers, professors, and future employers as part of their self-management. HCPs believed adolescents should learn to advocate for themselves to ensure they could succeed at school while ensuring the stress and schedule did not lead to a crisis. HCPs and parents emphasized that adolescents should know specific information and terms to use when going to the emergency department to effectively advocate for themselves and expedite their care, especially to HCPs who may be less knowledgeable about SCD.

##### Social Support

All 3 groups discussed the importance of social support and speaking to other adolescents with SCD. Adolescents found social support from their peers with SCD was beneficial in allowing them to talk about their experience with others who had similar experiences. Parents and adolescents described the benefits of a camp for kids with SCD and having a positive experience in terms of friendships and improvements in confidence. All groups mentioned the need for motivating role models who had SCD and had achieved success in some form. Adolescents and HCPs also believed older adolescents with SCD mentoring younger children would be helpful in terms of both social support and the transition process.

##### Information for Future Planning

All participants discussed the different impacts of SCD on future planning, and they recommended that challenging topics should be discussed early on so that adolescents could be given strategies to manage these issues. Adolescents mainly discussed the impact of SCD in choosing postsecondary education or training. Adolescents emphasized wanting to balance their ambitions with the realistic expectations of their disease as they grew older. Adolescents as well as HCPs described a need to inform adolescents about the genetics of the disease to ensure they understood the impact it could have on their future relationships and planning for children. HCPs believed this factor was important, especially given the lack of discussion about the disease among families. HCPs and parents believed education regarding risk-taking behaviors such as alcohol, drugs, and sex and its effects on SCD was important for adolescents to learn about. Parents were concerned about the adverse effects drugs could have with their disease and highlighting the importance of that to their children.

##### Transition Is Not One-Size Fits All

Adolescents’ thoughts on the transition to adult health services depended on their disease severity. Some adolescents felt less worried because they rarely had crises. Patients, parents, and HCPs were in agreement that the ideal time for transition would depend on the individual’s disease severity and cognitive abilities. Adolescents expressed interest in being taught how to manage their condition on their own such as making their own appointments and going to them alone. All participants emphasized that transition should be a gradual process.

#### Perceptions About Digital Self-Management Program

Adolescents, parents, and HCPs felt that a digital self-management program could be very useful, and they were unanimous in their suggestions on features for a digital self-management tool. Their recommendations for informational topics and features to include are summarized in Table 4.

##### Facilitating Transition Care

Adolescents and parents believed a digital self-management tool would be useful for transitioning to adult care as it could be used both independently and with families. Parents discussed the benefit of a website in cultivating independence in the adolescents because it could be a self-directed activity. All groups also described the benefits of a reliable resource that could be privately accessed by teens to learn about sensitive topics, as they may feel less comfortable speaking to HCPs and parents about those topics. HCPs believed such a program could be a medium to share these resources with families in an accessible way and provide educational resources for parents to foster self-management behaviors among their adolescents.

**Table 4:** Essential components of a digital sickle cell disease self-management program.

Format		Web-based or mobile app
**Educational content to include**		
	Medication	Dosages; mechanism of action; side effects; consequences of nonadherence; alternative options for treatment; interactions with drugs, alcohol, and other risk-taking behaviors; cost of medication and coverage by insurance; addiction, tolerance, appropriate use; and safe-keeping medications
	Information about SCD^a^	What is SCD?; genetics of SCD; what happens during a vaso-occlusive crises crisis?; symptoms and types of SCD (eg, milder types, pica, priapism, enuresis); treatment options; complications of SCD; latest research and developments in SCD; and information on risk-taking behaviors
	Preventing crises and pain management	Strategies to prevent vaso-occlusive crises (eg, keeping hydrated, limits to physical exertion, dressing in layers); how to manage stress in school?; what to do when a crisis comes on; how to make the most of being involved in school and activities without triggering a crisis; multimodal *3P* approach to pain management (psychological, physical, pharmacological); at what point should I come to the hospital if I have a crisis?; and advocating for appropriate treatment at hospitals
	Resources	Information about SCD organizations; social and financial supports available for SCD; available SCD-related education to families; and insurance information
	Communication	Self-advocacy; keeping up with schoolwork; communicating with peers about SCD; communicating with teachers, professors, and employers about SCD; and communicating with HCP^b^ about SCD
	Future	Guidance on pursuing successfully postsecondary education and careers; implications for relationships with others with sickle cell trait; and risk-taking behaviors and SCD such as drugs, alcohol, and sex
**Desired characteristics and features**		
	Cultural appropriateness	Addressing misconceptions about SCD; plain language for immigrant populations; and communicating with extended family and community members about SCD
	Social support	Forum or chat option to communicate with other youth with SCD; peer mentorship from older adolescents with SCD; and examples of role models
	Ask an HCP questions	Forum to ask questions; submit a question to be answered by an HCP; and live chat with an HCP
	Interactive and multimedia	Videos of youth explaining their experiences with SCD; visuals and diagrams; and games
	Safety and trustworthiness	Username and password-protected use; monitored social features; and evidence-based medical and practical information

^a^SCD: sickle cell disease.

^b^HCP: health care provider.

##### Accessibility

HCPs and parents both liked the accessibility of a digital self-management program, given the availability of the internet; adolescents and HCPs believed a website or mobile phone app were potential platforms. One parent described the accessibility a digital program could provide, especially for those with limited financial resources and time to attend in-person education sessions.

##### Effectively Engaging Adolescents and Families

Adolescents, parents, and HCPs all believed the digital self-management program needed to effectively engage youth and their families. Adolescents were less likely to use it if it was less engaging or too difficult to understand. All groups were in strong agreement about the need for a chat room or social support feature for adolescents to connect with peers with SCD to share their experiences and ideas and have emotional support from others going through similar experiences. Parents and adolescents had a concern about the safety of a digital program and ensuring any social features had a way to safeguard that only the intended users were accessing the program and ensuring the privacy of adolescents. Finally, participants described the benefit of a program helping to reduce the stigma associated with the disease by educating the public about the disease and preventing misconceptions about the disease.

## Discussion

### Principal Findings

To the best of our knowledge, this is the first study that examines the recommendations for a digital self-management and transitional care program for adolescents with SCD from the perspectives of adolescents, parents, and HCPs and re-examines the impact of SCD on adolescents. Participants described the psychosocial impacts of SCD and provided recommendations for self-management and transitional care to address these concerns. Adolescents, parents, and HCPs endorsed a digital platform as an effective and accessible way to deliver this information and provided recommendations for tailoring the program to adolescents with SCD. Adolescents focused on self-management experiences, especially those pertaining to school, friendships, and postsecondary education choices, whereas parents and HCPs focused on transitional care issues and sensitive topics.

Adolescents in this study described many of the psychosocial impacts of their disease, which pose challenges in disease self-management and transition to adult health care for this population. These findings support what has previously been described in studies with adolescents and adults with SCD. These include difficulties in developing peer relationships; limitations in physical activity; school and activity absenteeism; feelings of worry and uncertainty; disease-related stigma; and negative responses from peers, teachers, and HCPs [3,4,25-32]. These challenges were described by participants in the context of Ontario, Canada, and highlight the continued perceived challenges this population faces throughout childhood and adolescence. All participants highlighted the need for increased awareness of SCD among HCPs and school teachers, appropriate support for youth to succeed academically, and support to reduce the burden of the disease on families. Participants discussed the stigmatization of the SCD and several unique issues for the Canadian context. HCPs highlighted the SCD-related stigma among adolescents’ ethnic communities. Previous studies with families of children with SCD in African settings found varying levels of stigma toward SCD [33-36]. For families who immigrate to Canada from these nations or have ancestry from these nations, negative beliefs toward SCD may linger. To address these issues, participants in our study highlighted that awareness and communication were critical in addressing these impacts and could be delivered through a digital tool. Moreover, HCPs and parents in our study discussed a perceived lack of financial and practical supports and resources for SCD in Ontario, Canada. These findings appear to be reflected in a study examining the quality of information on SCD on the internet where none of the top 12 websites examined were of Canadian origin [37].

Adolescents, parents, and HCPs focused on empowerment through education and understanding SCD for self-management. The importance that adolescents placed on medical information is consistent with the qualitative study with youth with SCD and thalassemia by Atkin et al, which suggests that information is a resource that could offer a sense of control over adolescents’ lives, allowing them to develop preventative strategies and appropriate courses of action in response to crises [26]. This study extends those findings by identifying and articulating the specific topics of SCD information and self-management strategies needed for a digital self-management program.

Developing self-advocacy skills and communication strategies for communicating with peers, teachers, employers, and HCPs were highly emphasized by all groups as important in successful self-management and transition to adult health care. Similar emphasis on these skills has also been described by adults with SCD who shared their experiences in managing SCD in a qualitative study by Tanabe et al. Adults in that study highlighted that they often had to be their own advocates and make things work for themselves even with the limitations of the disease [38]. These recommendations are also in accordance with evidence showing that self-presentation and communication of condition to HCPs reduces stigmatization and allows for better care [28,39].

Adolescents in this study described self-management as a joint effort from everyone in their life; they sought support from friends, their families, and HCPs. They also described a need for social support from role models and peers with SCD. All groups discussed the importance of social support for dealing with negative emotions, feeling understood, and learning self-management strategies. Social support and role models as psychosocial interventions for SCD have been previously investigated as potentially effective interventions for adolescents with SCD [40-43]. These findings were consistent with views expressed by young adults in a qualitative study by Sobota et al examining the transitional care needs of young adults with SCD. Young adults in that study found that hearing about the transition process from someone who had already gone through it was more compelling [44]. Adolescents in this study believed a digital self-management program was an effective way for friends, peers, and families to learn about SCD and for them to connect with other youth with SCD.

All participants in this study preferred to learn about challenges and considerations they would face in the future regarding postsecondary education, choosing a career, and risk-taking behaviors (eg, drugs, sex) early on. Parents and HCPs believed information on risk-taking behaviors provided on a digital medium would make it easier for adolescents to access this information. Previous studies among youth with chronic diseases such as chronic pain or juvenile idiopathic arthritis identified similar information needs among adolescents and young adults [45,46]. This finding is also consistent with previous studies of adolescents’ preference for learning about health information on the Web, especially those regarding sensitive topics due to the anonymity offered by the internet [47,48]. All groups of participants in this study recommended a transitional care process beginning early in adolescence and tailored to the spectrum of SCD severity. They emphasized a need for a gradual process that allowed adolescents to take responsibility for specific tasks in their health care. These findings are well aligned with the findings of a systematic review of transition from adolescent to adult care for SCD, which recommended that transition programs be patient centric and flexible, and allow adolescents to explore independence and develop skills for managing SCD [49].

Development of a digital self-management and transitional care program tailored for adolescents with SCD was well endorsed by all participants. Parents, adolescents, and HCPs highlighted the accessibility of a digital program for families. HCPs believed that a potential mobile or Web-based program could be an acceptable format to be used by families and could help to address some of the stigmas among communities regarding the disease. Our findings are similar to those of a survey-based study conducted by Badawy et al regarding the mobile phone app preferences for medication adherence among adolescents with SCD. Our study extends the findings of Badawy et al by clearly describing in detail the important education topics to cover and the format in which youth preferred to receive social support in a comprehensive digital self-management program and the benefits they perceived [50]. Our study exemplifies the first step in effectively engaging patient and family users of digital interventions for chronic conditions by conducting a thorough investigation of their needs to understand their intended use and goal for these populations [51]. A systematic review of adolescents’ use of mobile phone and tablet apps for management of chronic conditions identified 3 of 4 studies that involved patient input in the development of the app [52]. However, only 1 of the 3 studies conducted an in-depth qualitative examination of the requirements articulated by patients and involved parents in the development process [52,53]. An earlier systematic review of the effectiveness of internet self-management interventions on health outcomes in adolescents with chronic conditions had similar findings, where interventions demonstrated limited involvement of parents [54]. Majeed-Ariss et al suggested that future interventions take into account social and family processes in their delivery and harness opportunities to promote shared self-management skills and knowledge [52].

### Limitations

The limitations of this study included the sample of participants who were recruited from a single tertiary care pediatric center and therefore limits generalizability of the findings. Individuals willing to participate in the interviews may be more motivated to manage their condition or have a more serious disease; this may limit the transferability of the results. This study was initially conducted in 2012; thus, perceptions and ideas about a potential digital self-management program may differ from the current time (eg, changes in delivery platform preferences). However, the focus of this study was on experiences and challenges related to adolescents managing SCD and the way in which a hypothetical digital program could assist in this self-management. Thus, any potential digital self-management program for adolescents with SCD should adhere to contemporary norms in the platform and interface design while being informed by the core components described by participants in this study. Furthermore, adolescent and parent participants were not offered an interviewer of the same sex or race as them, which may have prevented them from discussing more sensitive topics or speaking frankly with the interviewer. Atkin et al found that the sex of the interviewer may have been particularly important for adolescents while their previous work with parents of children with hemoglobinopathies found that race and sex were important characteristics of interviewers [26,55]. Finally, due to availability and timeline of the study, only a small sample of parents was included, with no male caregivers included, limiting the transferability of parents’ perspectives to other parents or caregivers.

In conclusion, participants in this study described the continued challenges youth with SCD face in Ontario, Canada, and provided recommendations for addressing these. Participants found value in developing a digital self-management program to address some of these challenges. Their perspectives on challenges and recommendations for self-management and transitional care will ensure the development of an acceptable and relevant digital program that meets the current needs of adolescents. Adolescents with SCD recommend the creation of a digital self-management program that provides in-depth SCD-related education in an accessible manner; fosters self-advocacy and communications strategies to be used in school, the workplace, and in health care settings; allows for social support; and provides an avenue to address challenging topics. This is the first study to identify the essential components of a digital self-management and transitional care program for this population and identify these requirements in relation to the psychosocial challenges that they face.

## Appendix

Multimedia Appendix 1 Themes and subthemes with exemplar quotations from participants.

## Abbreviations

CBT: cognitive behavioral therapy
HCP: health care provider
SCD: sickle cell disease
