# Supplementary material for: Exploring the Needs of Adolescents With Sickle Cell Disease to Inform a Digital Self-Management and Transitional Care Program: Qualitative Study
Source: JMIR Pediatr Parent. 2018 Sep 25;1(2):e11058. doi: 10.2196/11058 (PMC6716437; doi:10.2196/11058)
Supplement: Multimedia Appendix 1 [file pediatrics_v1i2e11058_app1.pdf]

## Multimedia Appendix

Themes and Sub-themes with Exemplar Quotations from Participants

| Self-management and Transitional Care Needs of Adolescents with SCD |
|---|
| (1) Impact of SCD |
| **Uncertainty of sickle cell disease**<br>“*I worry sometimes like… when I’m starting to get pain I’m like oh is this gonna escalate and am I going to have to go to the hospital*” (Adolescent #3, female, 14 years)<br><br>“*if I were them I think loss of control in my life cause I have no clue when this one is gonna come off… crisis pain, I have no idea when it’s gonna stop. I have no clue what is gonna happen with this admission cause each admission is different. So this uncertainty again and… loss of control, total loss of control on their lives*” (HCP Focus Group #3). |
| **Impact on developing peer relationships**<br>“*it affects how sometimes when I interact with my friend I guess because they’ll be doing stuff and then I’ll be totally left out cause I can’t do it like when they go swimming and stuff and I have to stay at home*” (Adolescent #9, female, 17 years)<br><br>“*It reminds them that they have this chronic disease that they’re gonna have for the rest of their life…and it makes them different from the rest of their peers and at a time when they’re just trying to fit in*” (HCP Focus Group #2) |
| **Academic impact**<br>“*I’m not there and I would miss whatever sometimes you don’t get marks on your report card etcetera but like I usually catch up… I make sure that I pay more attention because you know I missed this and even though I’m not even at school I’d call a friend ask what homework we’ve had for the past whatever my mom sometimes will pick up homework so like I could do it while I’m recuperating at home*” (Adolescent #1, male, 14 years)<br><br>“*I came out of the hospital and then I missed a lot and my teacher last year really didn’t understand so she gave me a project and I had to finish it in two days and it was a reading project so I had to read the book and I became really stressed out and I didn’t know what to do and I actually had pain again and I had to go back to the hospital*” (Adolescent #3, female, 14 years). |
| **SCD is stigmatizing**<br>“*I tell them I have sickle cell, they think it’s kind of weird cause they don’t understand it…they thought it was contagious so they all stood away from me for a long time…People don’t think it’s as serious…[they] think, oh you’re faking so you can get out of [school and work]…I had this teacher who’s like ‘oh [SCD] is not that bad…it doesn’t affect you that much’… Kids in my class [wouldn’t] take it that seriously cause the teacher kept saying it’s not a big* |

| |
|---|
| *deal"* (Adolescent #13, male, 13 years)<br><br>"*It's [almost] pathognomonic of black people and there's something probably always lurking at one level or another about the stigma and [disadvantaged and poorer community]…it's different than…CF or diabetes where you know the most famous people in the world have got it…here it's just one group…kids pick up on something like that and…in how some of us react to them [in the emergency department; ED] maybe because some of us have our prejudices"* (HCP Focus Group #4)<br><br>"*in Africa, having sickle cell is a bad thing… there's not many kids in certain parts that live till age two. So then you look at that and say, this woman had three or four kids who had sickle cell and they died by age two, she's looked upon as being an evil person because she had all these kids that died…where in the West Indies they have very good clinics and support so you don't die from sickle cell or few people die from sickle cell"* (HCP Focus Group #1). |
| (2) Experiences and challenges of self-management |
| **The Internet is a source of SCD-related education**<br>"*sometimes like I've tried to go on websites where you learn more about stuff and sometimes they just pile so many facts on it, it gets really, really boring and you really can't read through that much stuff and you're just like, okay I'm just gonna go off this"* (Adolescent #3, female, 14 years) |
| **Managing emergency department (ED) visits**<br>"[*they'll say*]'*they treat me as a drug seeker, they don't know what it's like to have this pain… they think I'm lying because I'm not crying but like my pain is very bad'* (HCP Focus Group #4) |
| **Self-management is a joint effort**<br>"*I would say everyone kind of has a part in it*" (Adolescent #8, female, 17 years)<br><br>"*…if I'm in the hospital and I'm missing out and I get upset and my friends call and, they tell me what I miss, I kind of feel better because I'm talking to them…[it] makes me feel like, okay I don't have to like worry*" (Adolescent #2, female, 14 years) |
| **Lack of system level supports**<br>"*I have to take a day off…when the children are sick in the hospital, it's into our income… cause sometimes you just don't have the days off at work or sometimes you have to call in [sick] because you just want to get paid…my husband works commission work so when the children are sick his attention at work is reduced so it affects us financially…there was a time when my manager said she couldn't give me the time off so that was also stressful emotionally*" (Parent #2).<br><br>*"On a grand scale…the cohesion and organization of sickle cell supports within Canada and for families…is abysmal at best. Certainly there are sickle cell associations but…it's not very easy to tap into for each family some of which are gonna be immigrant families, and it makes it just much more difficult. Often, their resources (financial and otherwise) are limited as* |

| |
|---|
| *well…[which] affects your ability to tap into social support systems"* (HCP Focus Group #1). |
| (3) Recommendations for self-management and transitional care |
| **Information to cope with and live with SCD**<br>"*I think the way to address that is to say 'Yes these are the facts we'll give you - we'll teach you skills to try and prevent them from happening and then if they do happen, which they will, despite doing that then we'll teach you ways that we can help manage it to make it more manageable and limit the impact it will have on your life'"* (HCP Focus Group #3). |
| **Self-advocacy and communication**<br>"*[having more information would help] to inform others cause I feel like every time I'm stuck telling people what it is I can't really explain it*" (Adolescent #8, female, 17 years)<br><br>"*stress, exam time okay then I need to do only two exams now and probably ask my professors to do two later on or whatever*" (HCP Focus Group #1) |
| **Social support**<br>"*cause your parents don't really- like you're the only person that has the sickle cell right your parents don't have it. So it would be easier to talk to someone that has the disease, I'd ask them questions that they'd be able to answer*" (Adolescent #4, female, 17 years) |
| **Information for future planning**<br>"*…for jobs and careers…it shouldn't mean that you should stop dreaming and going to the highest point and um having achievements…[but] you should watch out for things that may affect your sickle cell pain…if it's [a job that affects it], you might as well not take it because… every week you'll be like in the hospital…you won't actually get to work in that job so you won't get paid…you should set out realistic careers that you know in your body will be able to perform and live up to*" (Adolescent #13, male, 13 years).<br><br>"*I've been explaining to her you know that you need to understand that these drugs are bad yes these drugs are bad but if you take these drugs and you have sickle cell it's even worse, it can be deadly right*" (Parent #1). |
| **Transition is not one-size fits all**<br>"*it depends on the severity of the thing and it also depends on what help you would need*" (Adolescent #7, male, 18 years) |
| (4) Perceptions about digital self-management program |
| **Facilitating transition care**<br>"*I think things that they may be more hesitant to talk to parents or their health care team about…I think a website is really nice because nobody else has to know that you're reading about things that you have concerns about that are kind of private and maybe you don't feel so comfortable about asking*" (HCP Focus Group #1) |
| **Accessibility** |

| |
|---|
| “*I work every time I get an opportunity I work overtime…there are times that I can’t just afford to be going [to] extra things…but if I have something online while I’m doing my dishes, the laptop is on, it’s going… I can be educated as well*” (Parent #2) |
| **Effectively engaging adolescents and families**<br>“*[the website] would definitely get the message out and like with sickle cell… it’s not a bad thing it’s like it’s not a curse and whatever and it would be very useful actually so I agree with that I’m for it.*” (Adolescent #15, male, 19 years). |
